# A Well-Designed Implement for Promoting Population Health and Property *via* Insurance

**DOI:** 10.3389/fpubh.2021.766003

**Published:** 2022-01-31

**Authors:** Zhengqiao Liu, Li Zhao, Yang-Che Wu, Ming-Che Chuang, Mei-Chih Wang

**Affiliations:** ^1^Digital Economy Academy of Yango University, Fuzhou, China; ^2^Business School of Yango University, Fuzhou, China; ^3^Department of Finance, College of Finance, Feng Chia University, Taichung, Taiwan; ^4^Center for Chinese Social and Management Studies, Tunghai University, Taichung, Taiwan

**Keywords:** catastrophe insurance, public catastrophe insurance scheme, population health and property, minimum solvency requirement, default risk

## Abstract

The frequency and intensity of catastrophes (including natural disasters and pandemics) rise and damage the population's health, life and property more seriously. In order to protect population health and wealth via full insurance indemnity, many countries set up a public catastrophe insurance scheme (PCIS) to maintain the function of catastrophe insurance markets. Little literature discusses the smart payment way of contributions charged by PCIS. This article design a model to describe the upward trend and cyclic frequency and intensity of catastrophic events. Such characteristics also promote the business cycle of the insurance industry. We analyze the changes in catastrophic insurer's capital structures under three cases of that the volume-based charges to the PCIS may come from equity holders or policyholders or both. PCIS may entail a shift of equity capital toward minimum solvency requirements, and then adverse incentives regarding insurer's security level arise. Various numerical experiments illustrate the changes in equity position, default probabilities, or expected policyholder deficits. The results show that the payment way of contributions should be designed carefully, not only with regard to PCIS's finance balance but also the resultant incentives and effects.

## Introduction

Financial services include banking and insurance (1–3). The overall economic and commercial institutions have certain potential proliferation external economic effects (4, 5). Patrick (6) proposed two financial development hypotheses. When economic growth needs to be built on the basis of the healthy development of the entire financial system, it is called supply-led; while in the process of economic growth, the entire financial system is continuously stimulated and developed, which is called the demand-following hypothesis. Nishat and Saghir (7) put forward the “feedback hypothesis,” that is, financial development variables and economic growth variables operate causally in two directions at the same time. However, a key aspect of the financial market is the financial intermediary role of the insurance industry. Insurance market activities at the micro-level provide safety nets and protection for individuals and companies, while at the macro-level, funds raised from insurance premiums can be used by financial markets and have spillover effects on other financial markets (1, 8). Ward and Zurbruegg (5) and Hussels et al. (9) believe that companies can use the characteristics of insurance risk transfer and financing to promote economic growth. Therefore, these all highlight the importance of insurance activities to other sectors of the financial services industry. Many scholars have proposed supply-leading hypotheses (10–12) demand-following hypotheses (13–15), and feedback hypotheses (14, 16). Furthermore, some scholars believe that the insurance market and economic growth will vary according to different country conditions (10, 12) or that the size of the insurance market depends on the level of income (17).

However, the worldwide increasing frequency and intensity of disasters engender an upward trend in economic losses and insured losses. The weather-related disasters especially cause myriad harm such as hurricanes, floods, and droughts have increased over the past few years the Intergovernmental Panel on Climate Change, 2007 (18, 19). Twenty US insurance companies to go bankrupt after Hurricane Hugo 1989. Eleven became insolvent after Hurricane Andrew 1992. Twenty-seven insurers became insolvent between 2004 and 2011 after sequential hurricanes damaging Florida in 2004 and 2005 (20). Even worse, many low- and middle-countries have a significantly inadequate catastrophe insurance market. On the other hand, epidemics and pandemics have damaged humanity throughout recorded history, as shown in Table 1. The pandemic frequency over the past 300 years, roughly corresponding to the post-Industrial Revolution period, the outbreaks per 100 years increase twice as before (from 2 to 4) (21).

**Table 1:** Pandemics frequency since the middle ages.

**Period**	**Frequency**
1,300–1,400	1
1,400–1,500	1
1,500–1,600	4
1,600–1,700	1
1,700–1,800	5
1,800–1,900	3
1,900–2,000	5
2,000–2,021	3

A growing number of uninsured losses from catastrophes have forced the government in disaster-prone countries to find feasible solutions against such risk. There is no doubt that private insurers with capital constraints possibly go through hard periods, providing a possible rationale for government intervention. One form of public intervention is to develop a public catastrophe insurance scheme (PCIS), which offers a different trade-off between risk and return than the insurance products offered in the private market. Boulatov and Dieckmann (22) illustrated that the involvement of disaster insurance funds and the well-designed insurance in the catastrophe market could increase the demand in the private market. Many countries set up a kind of PCIS to manage Catastrophe risk. Some popular examples are listed in Table 2. Most schemes serve as reinsurers to provide the private insurance market with reinsurance such as Japanese Earthquake Reinsurance Co., Ltd., and Australian reinsurance pool corporation (ARPC). Some schemes are similar to insurers by offering primary insurance such as National Flood Insurance Program in the USA and Earthquake Commission in New Zealand. In addition to people's property, the agricultural industry is also exposed to the impact of Catastrophe such as crops and livestock. Abnormal weather strengthens the volatility of farm income. The governments thus devise programs and policies to help stabilize agriculture incomes, for example, the Turkish agricultural Insurance pool, Mongolia index-based livestock Insurance pool, and Thailand agricultural insurance pool. In addition, some developing countries are low-income households and have inadequate local catastrophe insurance markets, which lack capital and technical capacity to coverage throughout the whole country. The World Bank Group and the Global Facility for Disaster Reduction and Recovery (GFDRR) help such countries develop a regional risk pooling mechanism for managing catastrophic risks. Catastrophe risk pooling can bring in three advantages: lower reinsurance costs due to a better structured and diversified portfolio, joint reserves to retain the first aggregate loss, and economics of scale in operation. The lower capital and operating costs decrease the technical insurance premium. The popular pools are listed in Table 3. These pools not only assist governments and insurance regulators to enact regulatory and policy reforms but also provide reinsurance policies to local insurers.

**Table 2:** Comparison of typical natural catastrophe insurance schemes.

**Scheme name**	**Country**	**Year**	**Risk covered**	**Premium rate**
Catastrophe naturelles (CatNat)	France	1982	All natural disasters except windstorm, ice, and snow	Fixed rate
UK flood insurance regime	UK Gentlemen's Agreement	1960	Flood	Fixed rate
Earthquake commission (EQC)	New Zealand	1994	Comprehensive natural catastrophes	Fixed rate, 5‰
National insurance scheme	Australia	1984	Earthquake	Risk-based rate
Norsk naturskadepool	Norway	1980	Comprehensive natural catastrophes	Fixed rate
Florida hurricane catastrophe fund	USA	1993	Hurricane	Risk-based rate
California earthquake authority (CEA)	USA	1996	Earthquake	Risk-based rate
Taiwan residential earthquake insurance fund (TREIF)	Taiwan	2002	Earthquake	Fixed rate
National catastrophe insurance fund	Thailand	2012	Flood, earthquake, windstorms	Fixed rate

**Table 3:** Regional risk pooling mechanisms for managing catastrophic risks.

**Name of the fund**	**Year**	**Program**	**Insured countries**
The caribbean catastrophe risk facility (CCRIF) http://www.ccrif.org/	2007	Led by 16 countries in the Caribbean	Country shareholders
Europa re http://www.europa-re.com/	2008	Led by Southeast Europe (Albania, Serbia, Republic of Macedonia, Bosnia, Segovia, Montenegro)	Country shareholders
Asia catastrophe pool http://unfccc-clearinghouse.org/institutions/asia-catastrophe-pool	2007	Led by Asia Capital Re. Malaysia Sdn Bhd, (ACRM)	Pan-Asian markets, from the Middle East to China and Japan

*These funds have financial support from the World Bank Group*.

One financial goal of PCIS is that the scheme must strike a balance between its non-default and the demand for a limited fund from sponsors and donors. Some literature has proven the feasibility of long-term financial self-sufficiency of PCIS (19, 23) if the catastrophic losses follow an uptrend mean-reverting path. That is to say, PCIS only needs the initially limited fund for the establishment and then works well for a long time. The empirical experiment displays that such PCIS has a lower default rate and maintains its operation for a long time. However, these studies do not discuss some questions: whether the PCIS benefits policyholders and how the PCIS affects insurers' capital structure. Compared with the insurance guaranty fund, where (24) have shown that a self-supporting insurance guaranty fund may be beneficial for policyholders under the assumption of homogeneous firms and risk preference, no literature about PCIS discuss this issue. Our study will develop a theoretical framework to analyze the effect of the PCIS on policyholders' premiums and equity holders' capital.

One important consideration is how the PCIS charges contribution. Most PCISs charge fixed-rate contributions that are not directly linked to the insurer's insolvency risk. Such PCIS may cause adverse incentives for insurers and cross-subsidization between market players. If the PCIS will likely bail the insurers out after Catastrophes occur, the way will discourage the insurers from taking a protective approach against catastrophe risks such as the increase of equalization reserves and hazard spread. Compared with Cummins's affirmation (1988) that well-designed insurance guaranty funds should require risk-based premium payments to avoid adverse incentives, as my knowledge, no literature discusses how the payment way for contribution raises adverse incentives. The study will discuss this issue and derive a risk-based contribution for PCIS.

Some literature about insurance guaranty schemes has discussed the issue (25). However, there exist some differences between the insurance guaranty scheme and PCIS. Most insurance guaranty schemes do not include segments with property and casualty and are designed to bail out or take over the insurance companies on the verge of bankruptcy. The insolvency risk often comes from inner poor management or outer financial crisis or both. The premium of traditional insurance policy is calculated according to the assumption that the share of people claiming a loss should converge to a predictable average value when a sufficiently large number of policyholders are independently exposed to loss, i.e., the Law of Large Numbers holds. In the contrast, most PCISs serve as reinsurers. Some also issue catastrophe insurance policies through the sale of insurers. PCISs are designed to promote the local catastrophe insurance market against catastrophe risk by strengthening the capital capacity and the pricing ability of catastrophe insurance policies. Their insolvent risk often comes from unexpected super catastrophe events. Natural disasters often cause an accumulation of correlated claims. Thus, catastrophe risk invalidates the Law of Large Numbers (2012). Catastrophe loss distribution has three characteristics: fat tails, micro-correlations, and tail dependence (26). Such characteristics lead to non-diversification traps (27), which form an uninsured risk. Otherwise, catastrophe insurers need to charge high premiums so that exceed the expected loss, but the premium may be greater than what the insured is willing to pay.

The purpose of this article is to analyze which payment way of contributions is better off benefiting the catastrophe insurance market. We make some assumptions and set up three cases as follows: both shareholders and policyholders seek to maximize their advantage. The original stake equilibrium of policyholders and shareholders is based on a risk-adequate position. The PCIS is established as a non-profit organization with a self-financing goal. The PCIS ensures policyholders' full coverage and is funded through contributions computed by some fraction of insurers' premium income. The insurer directly pays the contribution (case 1), or the policyholder directly pays the contribution (case 2) or the policyholder pays the insurer full premium, and the insurer pays PCIS the contribution (case 3). Once the PCIS enters the catastrophe insurance market, the PCIS affects differently the policyholder's and shareholder's advantages under a different case. Then, their stake equilibrium will change as a different case. Furthermore, the value of assets is assumed to be a kind of fixed income securities with a fixed growth rate, and the value of liability (catastrophe losses) is assumed to follow a CIR process. We can get the closed-form solutions of present value for shareholder position, policyholder position, and insurers' default. The numerical analysis will show how the adverse incentive drives the change of stake balance changes under three cases.

The contributions of this paper are three-fold. First, to our best knowledge, the discussion on the smart payment way of contributions charged by PCIS is new to the related literature. The PCIS ensures full coverage to policyholders and charges the insurer contribution based on the fraction of the insurer's premium volume. The policyholders pay the insurer the premium for full insurance. Such allocation of premiums and contributions is crucial because it can stop a vicious cycle of reducing equity capital. Second, the study shows that the fair contribution needs to be based on the insurer's default risk. If the PCIS assigns different parameters α to insurers according to their default risk level, the insurers will have more incentive to improve their default risk by increasing the equity level. The payment way may lead to a virtuous cycle of risk management. Third, the insurer's asset value and liability value (catastrophe losses) are assumed to follow a fixed growth rate and a CIR process, respectively. We can get the closed-form value for the default put option. The various numerical confirm the conclusion in this study.

The remainder of the paper is organized as follows. Section The Contingent Claim Model for Stakeholder Positions offers the analysis of stakeholder positions in a contingent claim model. Stakeholder (policyholder, insurer, and PCIS) positions are modeled under three cases for payment ways of contributing to the PCIS. Section Numerical Experiment of the Impact on Stakeholder Positions carries out the numerical experiment of the impact on stakeholder positions. The value processes of assets and liabilities are modeled. We derive an analytical form of a default put option to measure insurers' insolvency risk. The numerical results are analyzed according to the assumptions of three cases and match the inferences in section The Contingent Claim Model for Stakeholder Positions. The final section makes the conclusion. The third case may be feasible. Insurers can raise their equity capital to improve the insolvency risk, and then negotiate less contribution rate with PCIS next year. The contribution rate (magnitude of volume-based levies) should be estimated carefully, not only with regard to PCIS's finance balance but also the resultant incentives and effects.

## The Contingent Claim Model for Stakeholder Positions

This section develops the contingent claim model based on that of Schmeiser and Wagner in 2013. However, there are some differences between PCISs and insurance guaranty schemes. Firstly, PICSs are designed to help those damaged by catastrophes such as the disaster refugee and the property and casualty insurance companies. Science technologies advance the prediction and model of catastrophes, which cause property and casualty insurers' liabilities. Insurance guaranty schemes bailout life insurance companies in individual financial distress. But, it is difficult to model and predict insurers' moral hazard and poor management which cause life insurers' liabilities. Secondly, insurance guaranty schemes charge life insurer contributions, but not all PCIS's contributions in this study come from property and casualty insurers. The supper catastrophe impacts insurers infrequently and heavily in 1 year, but the moral hazard and poor management accumulate the damage to insurers at least in sequent years. Therefore, we adopt a special model for catastrophe insured losses and special parameters for analyzing stakeholder positions.

The one-period contingent model framework in this study is assumed to be under complete and arbitrage-free markets. There are three stakeholders in the model: policyholders and equity holders of the insurance company, and following the introduction of the PCIS. The asset value and the liability value (catastrophe losses) are assumed to follow a fixed growth rate and a CIR process, respectively. The value of the default put option is adopted as a measure of the insurer's solvency (28). The initial balance of risk-adequate position of policyholders and equity holders is distributed due to the introduction of PCIS. The regulation requires the lower bound *E*^min^ of equity holder position for the solvency level. That is minimum capital requirements. If the insurer cannot achieve a fair return conditional on equity capital more than *E*^min^, they will give up the business.

### Stakeholder Positions Without PCIS

At the beginning of the period, *t* = 0, the insurance company's initial equity is denoted by *E*_0_, and policyholders pay the up-front premium (denoted by *P*_0_) to the insurer which grants the coverage of their claims on the position of the insured parties. Insurers' liabilities come from policyholders' claims and their value is denoted by *L*_0_. The initially available asset (denoted by *A*_0_) is the sum of *E*_0_ and *P*_0_, and can be invested in the capital market.

At the end of the period, at time *t*, the policyholders make the claims (denoted by *L*_*t*_) on the catastrophe insurance. If the available assets *A*_*t*_ exceed the liabilities *L*_*t*_, the policyholders get the indemnity *L*_*t*_. If not, the policyholders receive only the indemnity *A*_*t*_ which is the market value of the insurer assets. Assets *A*_*t*_ and liabilities *L*_*t*_ are assumed to vary stochastically. Therefore, the indemnity payment *P*_*t*_ from the insurers to the policyholders is given by


(1) $$\begin{array}{l} P_{t}=\min\left(L_{t},A_{t}\right)=L_{t}-{\left(L_{t}-A_{t}\right)}_{+} \end{array}$$

where (·)_+_ = max(·, 0). The equity position (denoted by *E*_*t*_) of shareholders is determined by the difference between the asset *A*_*t*_ and the liability *L*_*t*_


(2) $$\begin{array}{l} E_{t}=A_{t}-P_{t}={\left(A_{t}-L_{t}\right)}_{+} \end{array}$$

For studying fairness condition, we first denote the present values of the policyholder position, the liabilities, the equity position, and insurer' default by ${\text{H}}_{0}^{\text{P}}$ = PV[*P*_*t*_], ${\text{H}}_{0}^{L}$ = PV[*L*_*t*_], ${\text{H}}_{0}^{\text{E}}$ = PV[(*A*_*t*_–*L*_*t*_)_+_] and ${\text{H}}_{0}^{\text{DPO}}$ = PV[(*L*_*t*_–*A*_*t*_)_+_], respectively, where PV stands for the valuation operator. ${\text{H}}_{0}^{\text{E}}$ is like an equity call option with maturity *t* and strike price *L*_*t*_. ${\text{H}}_{0}^{\text{DPO}}$ means the present values of the insurer's default loss and is like a default put option with maturity *t* and strike price *L*_*t*_. According to Equation (1), ${\text{H}}_{0}^{\text{P}}$ can be expressed by


(3) $$\begin{array}{l} {\text{H}}_{0}^{P}=\text{PV}\left[L_{t}\right]-\text{PV}\left[{\left(L_{t}-A_{t}\right)}_{+}\right]={\text{H}}_{0}^{L}-{\text{H}}_{0}^{DPO} \end{array}$$

The initial premium *P*_0_ is fair or competitive for the policyholders if the net present value of the insured position is zero, that is to say,


(4) $$\begin{array}{l} P_{0}={\text{H}}_{0}^{P} \end{array}$$

The initial contribution is equal to the latter receiving a risk-adequate return. To be fair or arbitrage-free for the shareholders, the present value of the expected equity position must equal to the initial contribution *E*_0_, that is to say,


(5) $$\begin{array}{l} E_{0}={\text{H}}_{0}^{E} \end{array}$$

Equation (4) is equivalent to Equation (5) because of the assumption that the available asset *A*_*t*_ equals the sum of the equity position and policyholder position, i.e., *A*_*t*_ = *E*_*t*_ + *P*_*t*_. We have *P*_0_ = ${\text{H}}_{0}^{\text{P}}$ if and only if *E*_0_ = ${\text{H}}_{0}^{\text{E}}$ (Appendix A). Under such conditions of fairness for policyholder and shareholder, *E*_0_-*P*_0_ have infinite possible numbers because there are different values of ${\text{H}}_{0}^{\text{DPO}}$. From a shareholders' point of view, they can choose a specific equity level or solvency level to benefit themselves most as long as the equity endowment *E*_0_ is not less than minimum equity capital *E*^min^ required by solvency regulation.

### Three Cases for Payment Ways of Contribution to the PCIS

We first make some assumptions that are used in this study hereafter. There are three interested parties including shareholders, policyholders, and a public catastrophe insurance scheme (PCIS). The PCIS is a non-profit organization but needs to maintain a self-financing situation. Therefore, it charges ex-ante contributions (a kind of levy) from policyholders or shareholders or both to balance its financial revenue and expenditure. On the contract, both shareholders and policyholders seek to maximize their advantage by paying less and getting more under the same insurance protection or protection. We make some following assumptions during one-period *t*, and then analyze the effects of different origins of contribution on the changes in the net present value of three interested parties' positions. There are *N* policyholders whose average underwriting cost is ε_1_, and *n* insurers whose average underwriting cost is ε_2_. It is quite obvious that *N* is very larger than *n*. The government taxes insurers' profit by a rate τ. At time *t* = 0, let $E_{0}^{*}$ and $P_{0}^{*}$ denote the initial contributions of shareholders and policyholders, respectively. The available asset is *A*_0_ = *E*_0_ + *P*_0_-*Nε*_1_. In three settings of contribution origins, let $C_{0}^{*}$ denote the contribution to PCIS, and be calculated as a fraction α of the premium volume $P_{0}^{*}$, 0 ≦ α <1. The growth rate of asset value is assumed to be γ. At time *t*, the available asset is *A*_*t*_ = ${\text{e}}^{\gamma t}A_{0}$.

### Case 1: Insurers Pay the Contribution

The insurers pay the PCIS the ex-ante contribution $C_{0}^{*}$ directly to get the full protection of liability caused by issuing catastrophe policies but do not pass on it to their policyholders. That is to say, the insurers lose some profit to pay the levy in exchange for the limited capacity of the claim for the unlimited one. So, at time *t* = 0, the value of the insurer's asset decreases to


(6) $$\begin{array}{l} A_{0}^{*}=E_{0}^{*}+P_{0}^{*}-N\varepsilon_{1}-C_{0}^{*} \end{array}$$

Policyholders are not affected by the introduction of the PCIS. That is to say, $P_{0}^{*}$ = *P*_0_. In practice, policyholders are not aware of the existing default risk and the limited compensation of the insurers. This case is feasible when the higher premiums for full insurance offered by the insurers cannot be implemented in the market.

At time *t*, the insurers have the available asset $A_{\text{t}}^{*}$ = *A*_*t*_–*e*^γ*t*^$C_{0}^{*}$, where *A*_*t*_ = *e*^γ*t*^ ($E_{0}^{*}$ + $P_{0}^{*}$ –*Nε*_1_). The policyholders with claims *L*_*t*_ can get the indemnity $P_{t}^{*,I}$ from the insurers and the indemnity $P_{t}^{*,S}$ from PCIS if the insurers' asset had exhausted. The indemnity payment from the insurers to the policyholders is given by


(7) $$\begin{array}{l} P_{t}^{*,I}=\min\left(L_{t},A_{t}^{*}\right)=L_{t}-{\left(L_{t}-A_{t}^{*}\right)}_{+} \end{array}$$

The PCIS pays the remainder of claim *L*_*t*_. The indemnity payment is


(8) $$\begin{array}{l} P_{t}^{*,S}={\left(L_{t}-A_{t}^{*}\right)}_{+} \end{array}$$

The equity position is $E_{t}^{*}$ = $A_{t}^{*}$ – $P_{t}^{*,I}$ = ($A_{t}^{*}$–*L*_*t*_)_+_. The present value of the equity position is expressed as


(9) $$\begin{array}{l} {\text{H}}_{0}^{*,E}=\text{PV}\left[E_{t}^{*}\right]=\text{PV}\left[{\left(A_{t}^{*}-L_{t}\right)}_{+}\right] \end{array}$$

From the perspective of shareholders, when the PCIS is introduced, the net present value of the change in their equity position is:


(10) $$\begin{array}{l} {\text{H}}_{0}^{*,E}-E_{0}^{*}=PV\left[{\left(A_{t}^{*}-L_{t}\right)}_{+}\right]-PV\left[{\left(A_{t}-L_{t}\right)}_{+}\right]\\ \;=PV\left[{\left(A_{t}-L_{t}\right)}_{+}-\min\left(e^{\gamma }C_{0}^{*},{\left(A_{t}-L_{t}\right)}_{+}\right)\right]\\ \;-PV\left[{\left(A_{t}-L_{t}\right)}_{+}\right]\\ \;=-PV\left[\min\left(e^{\gamma }C_{0}^{*},{\left(A_{t}-L_{t}\right)}_{+}\right)\right]\in \left[-C_{0}^{*},0\right] \end{array}$$

The net present value is zero or negative. The zero value means that the initial investment $E_{0}^{*}$ is fair for shareholders or the situation with PCIS remains fair for the insurers. According to Equation (10), two extreme situations can lead to zero value. One is that the company becomes insolvent, i.e., (*A*_*t*_–*L*_*t*_)_+_= 0. Another is that the insurers pay no premium to PCIS, i.e., $C_{0}^{*}$= 0. Such two situations do not make any sense in Case 1. The shareholders actually face the negative value, and then promote the insurers to take any action against adverse conditions. They will lower their initial equity investment $E_{0}^{*}$ because that they need to decrease the negative value in Equation (10) and that the premium income $P_{0}^{*}$ is reduced by $C_{0}^{*}$ to (1–α) *P*_0_. That is to say, the equity position is adjusted to $E_{0}^{*}$ which is less than *E*_0_ without contribution $C_{0}^{*}$.

When we further consider the minimum equity level *E*^*min*^ required by solvency regulation, the lower $E_{0}^{*}$ may be larger or less than *E*^*min*^. If the latter one holds, the shareholders are not willing to support the issue of such catastrophe policies by sacrificing their equities. That is, the insurer will quit the catastrophe market. On the contrary, the former condition means that the insurers can offer the shareholders a fair return by adjusting the equity position. The catastrophe insurance will operate with lower equity $E_{0}^{*}$. The insolvency risk thus increases.

From the perspective of policyholders, they obtain the indemnity payments which consists of $P_{t}^{*,I}$ in Equation (7) and $P_{t}^{*,S}$ in Equation (8). Then, the present value of the policyholder premium, ${\text{H}}_{0}^{*,\text{P}}$, can be derived by


(11) $$\begin{array}{l} {\text{H}}_{0}^{*,P}=PV\left[P_{t}^{*,I}+P_{t}^{*,S}\right]=PV\left[L_{t}-{\left(L_{t}-A_{t}^{*}\right)}_{+}\right]\\ \;+PV\left[{\left(L_{t}-A_{t}^{*}\right)}_{+}\right]={\text{H}}_{0}^{L} \end{array}$$

According to Equations (3) and (4), the policyholder net present value is derived by


(12) $$\begin{array}{l} {\text{H}}_{0}^{*,P}-P_{0}^{*}={\text{H}}_{0}^{L}-\left({\text{H}}_{0}^{L}-{\text{H}}_{0}^{DPO}\right)={\text{H}}_{0}^{DPO}\geq 0 \end{array}$$

This means that the introduction of PCIS increases the policy value which equals the default put option of the insurer. In the setting, a rational policyholder in a transparent market requires lower premiums and does not care about the insolvency risk which reflects the value of the default put option. The competitive premium $P_{0}^{*}$ is adjusted decreasingly by reducing equity position and then contributes to a lower charge by the insurance scheme. However, policyholders' rights and claims are not affected whether the insurers go into bankruptcy or not.

From the perspective of PCIS, the present value of the PCIS payment $P_{t}^{*,S}$ to policyholders is denoted by ${\text{H}}_{0}^{*,\text{S}}$ = PV[$P_{t}^{*,S}$]. So, the net present value of PCIS position is given by


(13) $$\begin{array}{l} {\text{H}}_{0}^{*,S}+n\varepsilon_{2}-C_{0}^{*}=\text{PV}\left[{\left(L_{t}-A_{t}^{*}\right)}_{+}\right]+n\varepsilon_{2}-C_{0}^{*}={\text{H}}_{0}^{*,DPO}\\ \;+n\varepsilon_{2}-C_{0}^{*} \end{array}$$

A non-profit PCIS achieves a self-financing goal if and only if the net present value ${\text{H}}_{0}^{*,\text{DPO}}$ + *nε*_2_-$C_{0}^{*}$ is zero. If the value is negative, it means that the PCIS gets some profit. The PCIS can accumulate a reserve for the next period and then will lower the contribution $C_{0}^{*}$ due to the non-profit aim. On the contrary, the positive value will force the PCIS to raise the contribution $C_{0}^{*}$ in order to achieve its self-financed. The premium $C_{0}^{*}$ charged at time *t* = 0 should be ${\text{H}}_{0}^{*,\text{DPO}}$, which reflects the risk premium of insurer default. Therefore, the PCIS can adjust the value of $C_{0}^{*}$ by setting the parameter α based on the insolvency risk level of PV [(*L*_*t*_–A^*^ *t*)_+_].

In Case 1, when the insurers pay the contribution, the policyholders will lower their equity investment to reduce their negative net present value or to reflect their less premium income. The low equity will lead to high default risk. The policyholders do not care about such risk because the PCIS assures their full claim. When the PCIS affords more insurers' default risk and pays more value of ${\text{H}}_{0}^{*,\text{DPO}}$, PCIS will charge more contribution $C_{0}^{*}$ from the insurer by raising higher parameter α next period, and then the policyholders will lower their equity position again. This phenomenon will bring in a vicious cycle until either the equity reaches the minimum equity level *E*^*min*^ or the PCIS gets additional funding for supplementing the default (${\text{H}}_{0}^{*,\text{DPO}}$–$C_{0}^{*}$)_+_.

### Case 2: Policyholders Pay the Contribution

In this case, the policyholders pay the contributions to the scheme directly or indirectly through the insurers. Both insurers and shareholders are thus not affected by the contribution. At time *t* = 0, the initial equity $E_{0}^{*}$ equals to *E*_0_, and the up-front premium $P_{0}^{*}$ equals to *P*_0_. This implies that the insurers' available asset is


(14) $$\begin{array}{l} A_{0}^{*}=E_{0}+P_{0}-N\varepsilon_{1}=A_{0} \end{array}$$

At time *t*, $A_{0}^{*}$ equals *A*_0_ implies that A^*^ *t* equals *A*_*t*_. The policyholders can get two indemnities $P_{t}^{*,I}$ and contingent $P_{t}^{*,S}$. The indemnity payment $P_{t}^{*,I}$ from the insurer is


(15) $$\begin{array}{l} P_{t}^{*,I}=P_{t}=L_{t}-{\left(L_{t}-A_{t}\right)}_{+} \end{array}$$

Additionally, the indemnity payment $P_{t}^{*,S}$ from the scheme compensates for the default caused by policyholders' claims, which is the amount *L*_*t*_ over *A*_*t*_. That is to say,


(16) $$\begin{array}{l} P_{t}^{*,S}={\left(L_{t}-A_{t}\right)}_{+} \end{array}$$

From the perspective of shareholders, their equity position is $E_{t}^{*}$ = (*A*_*t*_–*L*_*t*_)_+_, and then the present value of the equity position is ${\text{H}}_{0}^{*,\text{E}}$ = PV[$E_{t}^{*}$] = PV[(*A*_*t*_–*L*_*t*_)_+_]. The change in equity position becomes


(17) $$\begin{array}{l} {\text{H}}_{0}^{*,E}-E_{0}^{*}=0 \end{array}$$

The above equation also reflects that shareholders are not directly affected by the introduction of the scheme and that they have a fair value of equity position.

From the perspective of policyholders, their initial position in *t* = 0 is $P_{0}^{*}$ = *P*_0_ + $C_{0}^{*}$ + *Nε*_1_. Because $C_{0}^{*}$ = α$P_{0}^{*}$, $P_{0}^{*}$ = *P*_0_ + *Nε*_1_ + α$P_{0}^{*}$ implies that the up-premium is:


(18) $$\begin{array}{l} P_{0}^{*}=\frac{1}{1-\alpha }\left(P_{0}+N\varepsilon_{1}\right) \end{array}$$

The policyholders can get full claims $P_{t}^{*}$ = *L*_*t*_ because of PCIS's assurance, and the present value of their position is the same as Equation (11), i.e.,


(19) $$\begin{array}{l} {\text{H}}_{0}^{*,P}={\text{H}}_{0}^{L} \end{array}$$

Then, using Equation (19) and Equations (3) and (4), the policyholder net present value is expressed by


(20) $$\begin{array}{l} {\text{H}}_{0}^{*,P}-P_{0}^{*}={\text{H}}_{0}^{*,L}-\frac{1}{1-\alpha }\left(P_{0}+N\varepsilon_{1}\right)\\ \;={\text{H}}_{0}^{L}-\frac{1}{1-\alpha }\left({\text{H}}_{0}^{L}+N\varepsilon_{1}-{\text{H}}_{0}^{*,DPO}\right)\\ \;=\frac{{\text{H}}_{0}^{*,DPO}-\alpha {\text{H}}_{0}^{L}-N\varepsilon_{1}}{1-\alpha } \end{array}$$

The fair condition for policyholders is that the net present value is zero if and only if the fraction α of their initial premium position is the ratio of (${\text{H}}_{0}^{*,\text{DPO}}$–*N*ε_1_) to ${\text{H}}_{0}^{\text{L}}$. We denote the ratio by the notation $\alpha_{policyholder}^{fair}$:


(21) $$\begin{array}{l} \alpha_{policyholder}^{fair}\equiv \frac{{\text{H}}_{0}^{*,DPO}-N\varepsilon_{1}}{{\text{H}}_{0}^{L}} \end{array}$$

where α^*fair*^ denotes the ratio. If α is larger than α^*fair*^, the situation is unfair for policyholders. They will require the PCIS to re-estimate insurers' default risk ${\text{H}}_{0}^{\text{DPO}}$ in order to lower the next-period contribution or look for low-premium policies, which are provided by the insurers with low equity, i.e., high ${\text{H}}_{0}^{*,\text{DPO}}$.

From the perspective of PCIS, it receives the contribution $C_{0}^{*}$ at time *t* = 0 and pays the claim $P_{t}^{*}$ at time *t*. The net present value of PCIS is expressed by


(22) $$\begin{array}{l} {\text{H}}_{0}^{*,S}+N\varepsilon_{1}-C_{0}^{*}={\text{H}}_{0}^{*,DPO}+N\varepsilon_{1}-C_{0}^{*} \end{array}$$

where ${\text{H}}_{0}^{*,\text{S}}$ = PV [(*L*_*t*_–*A*_*t*_)_+_]. The PCIS is self-financing if and only if the contribution $C_{0}^{*}$ equals to the value of insurer's default put option ${\text{H}}_{0}^{*,\text{DPO}}$+ *Nε*_1_. As $C_{0}^{*}$ = $\alpha P_{0}^{*}$, the self-financing condition $C_{0}^{*}$ = ${\text{H}}_{0}^{*\text{DPO}}$, implies α = (${\text{H}}_{0}^{*,\text{DPO}}$ + *Nε*_1_)/${\text{H}}_{0}^{\text{L}}$. We denote the ratio by the notation $\alpha_{PCIS}^{fair}$:


(23) $$\begin{array}{l} \alpha_{PCIS}^{fair}\equiv \frac{{\text{H}}_{0}^{*,DPO}+N\varepsilon_{1}}{{\text{H}}_{0}^{L}} \end{array}$$

If α is less than $\alpha_{PCIS}^{fair}$, the PCIS will raise the parameter α next period to maintain self-finance, and then the action will force policyholders to seek a lower premium to reduce their expenses in insurance. Compared with the one in Equation (21), $\alpha_{PCIS}^{fair}$ is larger than $\alpha_{policyholder}^{fair}$ because that the PCIS needs to pay the underwriting cost. The PCIS has a stronger pricing power than policyholders. The ratio $\alpha_{PCIS}^{fair}$ will be possibly adopted. In practice, ${\text{H}}_{0}^{\text{DPO}}$ is quite larger than *Nε*_1_. $\alpha_{policyholder}^{fair}$ is thus approximately equal to $\alpha_{PCIS}^{fair}$.

In this case, although the equity capital is fairly valued, policyholders pay the contribution based on the premium *P*_0_, and then have more motivation to find a low-premium policy. As a result of competitive pressure, the shareholders reduce their equity position to the minimum equity level *E*^min^ in order to offer a low-premium policy. This phenomenon will lead to a similar vicious cycle as that in Case 1 until either the equity reaches the minimum equity level *E*^min^ or the PCIS gets additional funding for supplementing the default (${\text{H}}_{0}^{*,\text{DPO}}$–$C_{0}^{*}$)_+_.

### Case 3: Policyholders and Insurers Pay the Contribution

In this case, the policyholders pay their insurers a premium $P_{0}^{*}$ calculated based on the full insurance, and then the insurers pay the PCIS the contribution $C_{0}^{*}$. The PCIS ensures full protection of the insurers' liability paid to the policyholders. In other words, $P_{0}^{*}$ is a risk-free premium ${\text{H}}_{0}^{\text{L}}$, and a fraction α, 0≦α < 1, of such premium, is transferred to the contribution $C_{0}^{*}$. The PCIS assures policyholders' full claim if the insurers run out of their assets. Because the condition is fair to the policyholders, the equality ${\text{H}}_{0}^{\text{L}}$ = *P*_0_ + ${\text{H}}_{0}^{\text{DPO}}$ holds according to Equations (3) and (4). At time *t* = 0, the insurer' available assets are given by


(24) $$\begin{array}{l} A_{0}^{*}=E_{0}^{*}+P_{0}^{*}-N\varepsilon_{1}-C_{0}^{*}=E_{0}+P_{0}-N\varepsilon_{1}+{\text{H}}_{0}^{*,DPO}-C_{0}^{*}\\ \;=A_{0}+{\text{H}}_{0}^{*,DPO}-N\varepsilon_{1}-C_{0}^{*} \end{array}$$

where the initial investments $E_{0}^{*}$ are assumed to be *E*_0_. $A_{0}^{*}$ is assumed to be invested in the same assets as *A*_0_. At time *t*, the indemnity payment $P_{t}^{*,I}$ from the insurer is equal to *L*_*t*_*-*(*L*_*t*_*-e*^γ*t*^$A_{0}^{*}$)_+_. The available asset based on Equation (24) is $A_{t}^{*}$ = *e*^γ*t*^$A_{0}^{*}$+(*L*_*t*_*-e*^γ*t*^ $A_{0}^{*}$)_+_. The shareholders' position at time *t* is $E_{t}^{*}$ = *e*^γ*t*^$A_{0}^{*}$ +(*L*_*t*_*-e*^γ*t*^$A_{0}^{*}$)_+_–*L*_*t*_. Additionally, the indemnity payment $P_{t}^{*,S}$ from the scheme compensates for the shortfall between insurers' payment and policyholders' claims. That is to say, $P_{t}^{*,S}$ = (*L*_*t*_*-e*^γ*t*^$A_{0}^{*}$)_+._

From the perspective of shareholders, by using similar transformations in Equations (9, 10), the net present value for shareholders is


(25) $$\begin{array}{l} {\text{H}}_{0}^{*,E}-E_{0}^{*}=-\text{PV}\left[\min\left(e^{\gamma t}C_{0}^{*}-{\left(L_{t}-A_{t}\right)}_{+},{\left(A_{t}-L_{t}\right)}_{+}\right)\right] \end{array}$$

where *A*_*t*_ = *e*^γ*t*^ ($E_{0}^{*}$ + $P_{0}^{*}$–*Nε*_1_), The derivation is listed in Appendix B. According to Equation (25), the condition for shareholders is fair if and only if the net present value is zero. $C_{t}^{*}$ thus equals (*L*_*t*_*-A*_*t*_)_+_. It implies that $C_{0}^{*}$ = PV[(*L*_*t*_–*A*_*t*_)_+_] = ${\text{H}}_{0}^{*,\text{DPO}}$, and shows that the fair contribution $C_{0}^{fair}$for shareholders is equal to the present value of the insurer's default put option. If $C_{0}^{*}$ is larger than ${\text{H}}_{0}^{*,\text{DPO}}$, the situation is unfair to shareholders. The insurers will either require the PCIS to reduce the contribution next period or decrease the equity capital to raise the default put value ${\text{H}}_{0}^{*,\text{DPO}}$. If the adjusted equity is still over the minimum equity level *E*^*min*^, the business will continue. Otherwise, the business disappears.

From the perspective of policyholders, because the PCIS grants full claims, the policyholders get the indemnity payment $P_{t}^{*}$ = *L*_*t*_. The net present value for policyholder equals zero because of the following equality


(26) $$\begin{array}{l} {\text{H}}_{0}^{*,P}-P_{0}^{*}=\text{PV}\left[P_{t}^{*}\right]-{\text{H}}_{0}^{L}=0 \end{array}$$

It is a fair situation for the policyholders in any α. The result is due that the policyholders pay the default-risk premium corresponding exactly to the full protection.

From the perspective of PCIS, the PCIS receives contributions via *n* insurers. It only face *n* insurers and spends underwriting costs *nε*_2_, which are quite less than *Nε*_1_ because *N* >> *n*. the net present value for PCIS can be written as ${\text{H}}_{0}^{*,\;S}$ + *nε*_2_ – $C_{0}^{*}$ = PV[(*L*_*t*_–*A*_*t*_)]_+_ + *nε*_2_ – $C_{0}^{*}$ = ${\text{H}}_{0}^{*,\text{DPO}}$ + *nε*_2_ – $C_{0}^{*}$. The PCIS is self-financing if and only if the contribution $C_{0}^{*}$ is equal to the value of the insurers' default put option ${\text{H}}_{0}^{*,\text{DPO}}$. The result is similar to the discussion in Case 1. However, both the assets A^*^ *t* and the contribution $C_{0}^{*}$, in this case, are larger than those in Case 1 because that the policyholders pay their insurers a premium supplement ${\text{H}}_{0}^{\text{DPO}}$ and that the contribution is charged by a fraction α of the default-free premium.

In this case, it is fair to the policyholders because they pay an ex-ante risk-adequate premium and get the full protection of the claim. Every policyholder pays the same premium and then the competitive premium will not be lowered under such that situation. This situation will avoid a vicious cycle of competitive pressure results in a race to the bottom, and then force the equity capital to the regulatory minimum equity level *E*^*min*^. On the contrary, whether this setting is fair to shareholders or PCIS depends on how the contribution $C_{0}^{*}$ is set on the parameter α. The high value of α favors PCIS but is unfavorable to policyholders. Conversely, if contrary. The fair condition for both shareholders and PCIS is $C_{0}^{*}$ = ${\text{H}}_{0}^{*,\text{DPO}}$ + *nε*_2_. Compared to policyholders, the PCIS has enough abilities and professional knowledge of assessing insurers' default risk and find the breakeven point


(27) $$\begin{array}{l} C_{0}^{fair}={\text{H}}_{0}^{*,DPO}+n\varepsilon_{2} \end{array}$$

If the equity position is larger than the regulatory minimum equity level *E*^*min*^ under such a fair situation, the business continues. Otherwise, the insurers will quit the catastrophe market.

### Overview of Three Cases and Further Discussion

This study makes assumptions of stakeholders: the PCIS assures insurers' liability or policyholders' claim, rational policyholders in a transparent market prefer the lowest premiums, and the shareholders tend to reduce their equity capital to gain policyholders. The shareholders in Case 1 pay the contribution and bear the negative net present value. They have more incentive to gain shareholders through high leverage (low equity capital). Simultaneously, the PCIS bears more insurers' default risk. That is to say, more value ${\text{H}}_{0}^{*,\text{DPO}}$ which benefits the policyholders. In Case 2, policyholders pay the contribution based on the premium *P*_0_, and then have more incentive to find a policy with a low premium. Competitive pressure results in a race to the bottom. Both Cases 1 and 2 easily lead to bring in a vicious cycle until either the equity position drops to the minimum equity level *E*^*min*^ or the business is discontinued. However, in Case 3, all policyholders pay the insurers the same premium for full insurance. The way avoids a premium race to the bottom. The PCIS charges the contribution $C_{0}^{*}$ from the insurers. The insurers maybe want to gain the shareholders by reducing equity capital, i.e., raising financial leverage. But, the PCIS can find the breakeven point $C_{0}^{fair}$= ${\text{H}}_{0}^{*,\text{DPO}}$ + *nε*_2_ by exactly assessing insurers' default risk.

Setting a unique α for market participants means to assume that all insurers are homogeneous. This condition is unfair to insurers with high equity positions (good solvency or low default risk) and then leads to adverse selection in the catastrophe insurance industry. If the PCIS assigns different parameters α to insurers according to their default risk level, the insurers charged high contribution will have more incentive to improve their default risk. They can require PCIS to lower the contribution by showing their high solvency. This way may lead to a virtuous cycle of reducing default risk.

## Numerical Experiment of the Impact on Stakeholder Positions

This section carries out numerical experiments and analyzes the results. According to the abovementioned stakeholder framework, the positions of stakeholders are evaluated under three settings. The changes in policyholders' premium, equity holders' position, and scheme fund's position are analyzed to make sure the inferences in the above section. Adverse incentives discussed can bring a huge impact on stakeholders.

In the following numerical analysis, we assume the market values of assets *A*_*t*_, equity capital *E*_*t*_, policyholder premium *P*_*t*_, PCIS contribution *C*_*t*_, and liability *L*_*t*_ for time *t*. Assets and liabilities are both modeled stochastically. After modeling the stakeholder positions, we derive closed-form solutions and then carry out a numerical simulation to illustrate the effects of a PCIS on the different stakeholder positions under different payers of contribution.

Closed-form solutions exist for the values of insurers' default, equity holder claims, and policyholder claims in the case without PCIS. Most insurers' capital is invested in stable assets due to the statute of limitation. To reducing the complexity of the model, the insurers' asset *A*_*t*_ is assumed to just meet the minimum requirement of risk-adjusted capital and are invested in fixed income securities with yield rate γ. However, the catastrophe events have the cyclicality and extreme nature. The Cox–Ingersoll–Ross square-root model [(29), CIR] is shown to be the best-fitting model for the time series of insured losses (19, 23). We thus define the asset dynamic *A*_*t*_ and the loss dynamic *L*_*t*_ as follows:


(28) $$\begin{array}{l} dA_{t}=\gamma A_{t}dt \end{array}$$

(29) $$\begin{array}{l} dL_{t}=a\left(b-L_{t}\right)dt+\sigma_{X}\sqrt{L_{t}}dW_{L,\;t} \end{array}$$

where *L*_*t*_ follows a CIR model. The term *a* (*b*–*L*_*t*_) defines a mean-reverting drift pulling the insured losses toward the long-term growth rate *b* with an adjustment speed *a*. σ_*X*_ denotes the instantaneous volatility rate of *W*_*L*,*t*_. Given an information set, *Y*_*T*_:=*c*_*T*_*L*_*T*_ follows a non-central Chi-Square distribution:


(30) $$\begin{array}{l} Y_{T}:=c_{T}L_{T}∽\;\chi_{nc}^{2}\left(n,\delta_{T}\right),\;g_{Y}\left(\phi \right)=\frac{\exp\left(\frac{i\phi \delta_{T}}{1-2i\phi }\right)}{{\left(1-2i\phi \right)}^{\frac{n}{2}}} \end{array}$$

where *g*_*Y*_(ϕ) is a characteristic function of *Y*.


(31) $$\begin{array}{l} c_{T}=\frac{4a}{\sigma^{2}\left[1-\exp\left(-a\left(T-t\right)\right)\right]},n=\frac{4ab}{\sigma^{2}},\delta_{T}\\ \;=c_{T}L_{T}\exp\left[-a\left(T-t\right)\right],2ab>\sigma^{2} \end{array}$$

From the perspective of option' payoff, the present value of the equity holder position, ${\text{H}}_{0}^{\text{E}}$ = e^−γ*t*^*E*^Q^ [(*A*_*t*_–*L*_*t*_)_+_], corresponds to the value of the put option with underlying price *A*_*t*_ and strike price *L*_*t*_. The value of European put option is obtained by the following expectation:


(32) $$\begin{array}{l} {\text{H}}_{t}^{E}=E_{t}^{Q}\left[e^{-r\left(T-t\right)}\left(K-L_{T}\right)I_{\left\{0<L_{T}<K\right\}}\right]\\ \;=Ke^{-r\left(T-t\right)}\cdot \left(1-\Pi_{2,c_{T}K}\right)-\frac{n+\delta_{T}}{c_{T}}e^{-r\left(T-t\right)}\left(1-\Pi_{1,c_{T}K}\right) \end{array}$$

where


(33) $$\begin{array}{l} \Pi_{1,d}^{p}=\frac{1}{2}+\frac{1}{\pi (n+\delta_{T})}\text{Re}\left(\int_{0}^{\infty }\frac{e^{-i\phi d}{g^{\prime }}_{Y}(\phi )}{i\phi }d\phi \right),\Pi_{2,d}^{p}\\ \;=\frac{1}{2}+\frac{1}{\pi }\text{Re}\left(\int_{0}^{\infty }\frac{e^{-i\phi d}g_{Y}(\phi )}{i\phi }d\phi \right) \end{array}$$

*K* = *A*_0_e^γ(*T*−*t*)^. *I*_{*event*}_ is an indicator function which equals 1 if the event occurs, otherwise, it is 0. *g*_*Y*_(ϕ) is the characteristic function of the CIR model with parameter ϕ. Re(·) denotes the real part of a complex number (see Appendix A).

Analogously, the present value of the expected policyholder claim is expressed by:


(34) $$\begin{array}{l} {\text{H}}_{0}^{P}=e^{-rt}E^{Q}\left[L_{t}\right]-e^{-rt}E^{Q}\left[{\left(L_{t}-A_{t}\right)}_{+}\right] \end{array}$$

which corresponds to the value of the call option with underlying price *L*_*t*_ and strike price *A*_*t*_. This term e^−γ*t*^*E*^Q^[(*L*_*t*_ –*A*_*t*_)_+_] is the value of default put option ${\text{H}}_{0}^{*,\text{DPO}}$ from the insurer, and the value can be obtained by the analytical pricing formula call options (30) as follows:


(35) $$\begin{array}{l} {\text{H}}_{0}^{*,DPO}=E_{0}^{Q}\left[e^{-r}\left(L_{1}-A_{0}e^{\gamma }\right)I_{\left\{A_{0}e^{\gamma }<L_{1}\right\}}\right]=E_{0}^{Q}\left[L_{1}\right]e^{-r}\Pi_{1}^{c}\\ \;-A_{0}e^{\gamma -r}\Pi_{2}^{c} \end{array}$$

where


(36) Π1c=12+1π∫0∞Re(A0e-iϕγg(ϕ-i)iϕg(-i))dϕ,Π2c        =12+1π∫0∞Re(A0e-iϕγgL(ϕ)iϕ)dϕ

$E_{0}^{Q}\left[L_{1}\right]=\frac{n+\delta_{1}}{c_{1}}$ (according to Appendix A).

### Equity Holder Capital Adjustment in Case 1

This section focus on the values of equity holders ${\text{H}}_{0}^{*,\text{E}}$ and policyholders ${\text{H}}_{0}^{*,\text{P}}$ by using the same framework and assumptions introduced in this section The Contingent Claim Model for Stakeholder Positions. Since the insurer contributes $C_{0}^{*}$ to the PCIS without passing on charges to the policyholders, an unfavorable situation with negative net present value happens to equity holders [see Equation (10)]. We focus on their incentives and reactions. The asset value decreases to $A_{0}^{*}$ = *A*_0_–$C_{0}^{*}$. The equity value ${\text{H}}_{0}^{*,\text{E}}$ is derived from Equation (32):


(37) $$\begin{array}{l} {\text{H}}_{0}^{*,E}=\text{PV}\left[E_{1}^{*}\right]=\text{PV}\left[{\left(A_{1}^{*}-L_{1}\right)}_{+}\right]=K^{*}e^{-r\left(T-t\right)}\left(1-\Pi_{2,c_{T}K}\right)\\ \;-\frac{n+\delta_{T}}{c_{T}}e^{-r\left(T-t\right)}\left(1-\Pi_{1,c_{T}K}\right) \end{array}$$

where K^*^ = $A_{0}^{*}$e^γ(*T*−*t*)^. Finally, since the policyholder can get full claim due to the introduction of PCIS, the values of the policyholder claims are obtained from Equation (11):


(38) $$\begin{array}{l} {\text{H}}_{0}^{*,P}=E^{Q}\left[e^{-rt}L_{t}\right]=L_{0} \end{array}$$

For purpose of the numerical experiment, the CIR model is calibrated via the global insured losses from 1970 to 2016. Table 4 shows the descriptive statistics of global insured losses. These 47 insured losses vary from a minimum of 5.5678 to a maximum of 147.3440 (billion USD). The standard deviation of 28.2100 is less than the mean of 33.0018. The data distribution has more right skewness of 2.3548 and a much higher kurtosis of 6.8158 than the normal distribution. The augmented Dickey-Fuller (ADF) test statistic rejects the null hypothesis of a unit root. It also reflects that the time series loss data are stationary. The maximum likelihood estimation is used to estimate three parameters in the CIR model, which are listed in order: the adjustment speed *a* = 0.9354, the long-term growth rate *b* = 33.6811, and the instantaneous volatility rate σ_*X*_ = 5.7062. The riskless rate *r* is assumed to be 0.03. Because the insurance industry covered close to USD 54 billion in 2016 (31), we take 54 as the initial liabilities *L*_0_. The yield rate γ of fixed income securities is set to be 0.05. The PCIS charges 1 percent of the policyholder premiums *P*_0_. That is to say, the contribution rate α equals 0.01. The value of such a rate is chosen from the one used in practice (25).

**Table 4:** Descriptive statistics of global insured losses adjusted in 2016 prices from 1970 to 2016.

	**Number**	**Mean**	**Median**	**Standard deviation**	**Minimum**	**Maximum**	**Skewness**	**Kurtosis**	**J-B**	**ADF**
Insured losses	47	33.0018	24.5308	28.2100	5.5678	147.3440	2.3548	6.8158	110.8799 (0.001)	−2.5375 (0.0125)

*The values of global insured losses, in billions of US dollars, are collected from the Sigma journal, through Statista's database, issued by Swiss Re every year, and are adjusted by the US consumer price index for December 2016. The J-B denotes the test statistic for the Jarque–Bera normality test with two degrees of freedom. The ADF stands for the augmented Dickey-Fuller test with the null hypothesis of a unit root. The p-values are indicated in parentheses*.

We take an example to explain the process of numerical experiments. For simplifying the model, we ignore the term *Nε*_1_ and *nε*_2_ because they are quite less than $P_{0}^{*}$, $E_{0}^{*}$ and ${\text{H}}_{0}^{*,\text{DPO}}$ in the real world. First, the initial default put option ${\text{H}}_{0}^{*,\text{DPO}}$ is set to be 1. That is to say, the initial insurer's safety is fixed. According to Equation (3), the initial premium *P*_0_ needs to be 53. The initial asset *A*_0_ is thus estimated when ${\text{H}}_{0}^{*,\text{DPO}}$ = 1 is substituted into Equation (35). Then, we can get the equity value ${\text{H}}_{0}^{*,\text{E}}$ = 64.4662 by instituting the value of asset *A*_0_ = 117.4662 into Equation (35). If an insurer joints the PCIS, the scheme charges the insurer the contribution $C_{0}^{*}$ = 0.99 if the contribution rate α = 0.01. The total assets after paying the fund contribution are $A_{0}^{*}$ = *A*_0_-$C_{0}^{*}$ = 116.4762. The insurer's solvency position thus decreases when its liability is not unchanged. The present value of the default put option ${\text{H}}_{0}^{*,\text{DPO}}$ will increase to 1.5655 by instituting the initial asset $A_{0}^{*}$ = 116.4762 into Equation (34). It means that the PCIS bear more insolvency risk and then needs to charge the insurer more contribution. From equity holders' point of view, their position changes from 64.4662 to 63.0161 when the asset value 117.4662 drops to 116.4762, and is not fair because the net present value drops to −1.4501. They will be incentivized to reduce the next-year equity capital from 64.4662 to 55.3022 in order to result in a fair situation. The reasons are that the asset value drops to 116.4762 and ${\text{H}}_{0}^{*,\text{DPO}}$ value increases to 1.5655. The PCIS will adjust the contribution rate α to maintain its finance. Such a situation brings in a vicious cycle of forcing the equity capital to the regulatory minimum equity level *E*^min^.

Table 5 illustrates multiple premium *P*_0_-equity holder capital ${\text{H}}_{0}^{*,\text{E}}$–the default put option ${\text{H}}_{0}^{*,\text{DPO}}$ combinations at equilibrium. Less premium *P*_0_ increases the value of the default put option ${\text{H}}_{0}^{*,\text{DPO}}$, and reduces the equity holder capital ${\text{H}}_{0}^{*,\text{E}}$. The PCIS incentivizes the combination. Lager contribution rate α under the same premium *P*_0_ increases the value of the default put option ${\text{H}}_{0}^{*,\text{DPO}}$, and quickly drops the equity holder capital ${\text{H}}_{0}^{*,\text{E}}$ to the regulatory minimum equity level *E*^min^.

**Table 5:** Adapted equity holder capital and default put option before and after introducing PCIS with insurer paying contribution.

**Policyholder premium**	Equity holder capital ${\text{H}}_{0}^{*,E}$, default put option ${\text{H}}_{0}^{DPO}$		
$P_{0}^{*}$ = *P*_0_	**Without PCIS α = 0**	**α = 1%**	**α = 3%**
53	1, 64.4662	1.5655, 55.3022	2.7665, 43.9594
52	2, 49.5214	2.5751, 44.5472	3.7911, 37.1735
51	3, 40.8138	3.5832, 37.4736	4.8111, 32.1141
50	4, 34.7158	4.5896, 32.2523	5.8271, 28.1170
49	5, 30.0699	5.5948, 28.1542	6.8393, 24.8429
48	6, 26.3538	6.5987, 24.8121	7.8480, 22.0930
47	7, 23.2846	7.6016, 22.0148	8.8535, 19.7413
46	8, 20.6932	8.6033, 19.6294	9.8555, 17.7033
45	9, 18.4686	9.6039, 17.5663	10.8549, 15.9170
44	10, 16.5356	10.6036, 15.7625	11.8513, 14.330
43	11, 14.8388	11.6022, 14.1716	12.8450, 12.9352
42	12, 13.3384	12.6001, 12.7589	13.8360, 11.6799
41	13, 12.0031	13.5969, 11.4978	14.8245, 10.5520
40	14, 10.8087	14.5930, 10.3662	15.8105, 9.5349
39	15, 9.7362	15.5883, 9.3474	16.7941, 8.6150
38	16, 8.7698	16.5827, 8.4275	17.7755, 7.7809
37	17, 7.8965	17.5764, 7.5948	18.7547, 7.0233
36	18, 7.1057	18.5693, 6.8394	19.7317, 6.3339
35	19, 6.3882	19.5616, 6.1532	20.7066, 5.7059
34	20, 5.7368	20.5532, 5.5291	21.6797, 5.1332

### Fairness of Total Premium in Case 2

Using the same framework and assumptions introduced early in section Numerical Experiment of the Impact on Stakeholder Positions, we focus on the values of policyholders ${\text{H}}_{0}^{*,\text{P}}$ under different initial premiums *P*_0_ and contributions rates α because the situation of the equity holders is not altered due to policyholders' paying PCIS contribution. The total initial premium $P_{0}^{*}$ paid by policyholders is expressed as a function of contribution rate α (for PCIS) and the initial premium *P*_0_ (for insurer): $P_{0}^{*}$ = *P*_0_/(1–α) [see Equation (19)]. The fair present value of the final payoff is given by $P_{0}^{*}$ = ${\text{H}}_{0}^{*,\text{P}}$ = ${\text{H}}_{0}^{\text{L}}$ [see Equation (20)] when the policyholder claims are guaranteed fully. Without loss of generality, we assume a present value $H_{0}^{L}$ of claims *L*_0_ of 54. Then, the fair initial total premium $P_{0}^{*}$ paid by policyholders is equal to 54.

In Table 6, we illustrate the policyholder total premiums for three initial premiums of *P*_0_= 53, 43, and 33. The case of initial premium *P*_0_ = 53 with α = 1.9% corresponds to a fair situation. However, α > 1.9% is unfair for policyholders due to the more total premium $P_{0}^{*}$. On the contrary, α < 1.9% is unfair for PCIS due to less contribution $C_{0}^{*}$. Similarly, the case of initial premium *P*_0_ = 43 (32) with α = 2.3% (2.7%) corresponds to a fair situation for both policyholders and PCIS. The low initial premium means the PCIS paying more claims from the insurer's default. Then, it is necessary for PCIS to charge more contributions.

**Table 6:** Policyholder total premium $p_{0}^{*}$ for different contribution rates α and initial premium *p*_0_ in policyholder paying contribution.

**Contribution rate**	Policyholder total premium $P_{0}^{*}$		
**α (in %)**	***P*_0_ = 53**	***P*_0_ = 43**	***P*_0_ = 33**
0	53	43	33
0.0020	53.1062	43.0862	33.0661
0.0040	53.2129	43.1727	33.1325
0.0060	53.3199	43.2596	33.1992
0.0080	53.4274	43.3468	33.2661
0.0100	53.5354	43.4343	33.3333
0.0120	53.6437	43.5223	33.4008
0.0140	53.7525	43.6105	33.4686
0.0160	53.8618	43.6992	33.5366
0.0180	53.9715	43.7882	33.6735
0.0200	54.0816	43.8776	33.6735
0.0220	54.1922	43.9673	33.7423
0.0240	54.4148	44.0574	33.8115
0.0260	54.5267	44.1478	33.8809
0.0280	54.6392	44.2387	34.0206
0.0300	54.7521	44.4215	34.0909
0.0320	54.8654	44.5135	34.1615
0.0340	54.9793	44.6058	34.2324
0.0360	55.0936	44.6985	34.3035
0.0380	55.2083	44.7917	34.3750
0.0400	55.3236	44.8852	34.4468

Although the uniform contribution rate α for different initial premiums *P*_0_ is always unfair for either policyholders or PCIS, the mandatory PCIS, in practice, charges some fixed contribution rate α to policyholders (as shown in Table 2) due to easy implementation and political consideration. Policyholders do not care about the insurer's safety due to PCIS's guaranty and tend to choose the policy with offering the lowest premium. The insurer thus lowers the equity capital to maintain its profit and then leaves more insolvency risk to PCIS.

### Safety Level of Solvency in Case 3

In this subsection, we focus on equity holder capital $E_{0}^{*}$ in different contribution rates α because policyholders pay the insurer uniform premium $P_{0}^{*}$ = ${\text{H}}_{0}^{\text{L}}$ for full coverage *L*_1_. The insurer contributes the PCIS according to different contribution rates α based on the insurer's insolvency level $C_{0}^{*}$ = ${\text{H}}_{0}^{*,\text{DPO}}$ [see in Equation (27)]. We assume a present value ${\text{H}}_{0}^{\text{L}}$ of claims *L*_0_ of 54 again. That is to say, $P_{0}^{*}$ = 54. The initial asset *A*_0_ is estimated when ${\text{H}}_{0}^{*,\text{DPO}}$ = $C_{0}^{*}$ is substituted into Equation (35). $E_{0}^{*}$ can be obtained by the initial asset *A*_0_ substituted into Equation (37). On the contrary, if equity holders raise their capital, the less value of ${\text{H}}_{0}^{*,\text{DPO}}$ is obtained by the more asset *A*_0_ substituted into Equation (35). The insurer can negotiate the fair contribution rate with the PCIS by showing its lower solvency risk (smaller value of ${\text{H}}_{0}^{*,\text{DPO}}$). Comparing with adjusting the contribution rate to different policyholders with different premiums, the adjustment of the contribution rate based insurers' insolvency risk is quick feasible because that the number of the insurer is far less than the number of policyholders and that both PCIS and insurers have enough professional knowledge to make a fair contribution rate.

Table 7 illustrates the importance of the choice of α with regard to an insurer's target equity capital. It lists that a variation of α from 0.0001 to 0.05 makes the fair equity capital vary from 151.1501 to 138.5996. As the contribution rate rises, the fair equity capital drops. On the contrary, the increase of equity holder capital may convince the PCIS of decreasing the contribution. The illustration in Table 7 shows that policyholders and insurers paying contributions may lead to a virtuous cycle of reducing default risk.

**Table 7:** Fair capital of equity holder $E_{0}^{*}$ for different contribution rates α in policyholder and insurer paying contributions.

**Contribution rate α**	**Default put option ${\text{H}}_{0}^{DPO}$**	Equity holder capital ${\text{H}}_{0}^{*,E}$
0.0001	0.0141	151.1501
0.0005	0.0269	138.5996
0.001	0.0543	124.7813
0.005	0.2700	91.9504
0.01	0.5400	77.1096
0.02	1.0800	61.7813
0.03	1.6200	52.5649
0.04	2.1600	45.9076
0.05	2.7000	40.6851

### A Risk Measure Illustration for Policyholders Paying' Full Insurance Premium and Insurers' Paying Contribution

In order to further detail the direct influence of PCIS on the solvency level, this subsection gives a parameterization example of policyholders and insurers' paying contribution with initial liability *L*_0_ = 33 and initial premiums *P*_0_ = 33 because that the mean of global insured loss from 1970 to 2016 is 33.0018 and that the insured get full coverage in this case. The objective is to calculate changes in the insurer's default probability and the expected policyholder deficit when equity holders adjust their capital to get back to even.

Table 8 shows that the extreme variations arise before and after the PCIS intervenes in the catastrophe market. The fair equity capital endowment reduced by 0.35% from *E*_0_ = 88.1200 to $E_{0}^{*}$ = 57.3657 when the insurer is charged by a contribution rate α = 0.01. Simultaneously, the available assets decrease by a quarter from *A*_0_ = 121.0800 to $A_{0}^{*}$ = 90.0357. The default probability increases more than eight times from 0.17 to 1.4207%. The expected policyholder deficit increases more than seven times from 0.03 to 0.2213. The result shows the significant immediate impact of the introduction of the PCIS on capital structure.

**Table 8:** Shortfall probability and expected policyholder deficit before and after introducing PCIS in insurer and policyholder paying contributions.

	**PCIS in insurer and policyholder paying contribution**			
**Item**	**α = 0**	**α = 0.5%**	**α = 1%**	**α = 2%**
Equity holder capital	88.1200	68.1592	57.3657	46.4538
Available assets	121.0800	100.9942	90.0357	78.7938
Default probability (in %)	0.1700	0.6874	1.4207	2.9230
Expected policyholder deficit	0.0300	0.1045	0.2213	0.4677

From the perspective of policyholders, it seems disadvantageous to introduce PCIS due to the increase of the expected deficit. However, the PCIS does not default and guarantees policyholders full coverage. Although the expected deficit with PCIS is higher than that without PCIS, the peak risk of heavy losses has been smoothened. Without PCIS, policyholders may suffer heavy losses and cannot get sufficient claims because of the insurer's default once a large Catastrophe occurs. The infrequent peak risk may force the insured into financial distress.

Furthermore, from the perspective of the business cycle, whether pro-cyclical or reverse-cyclical overall economic factors will affect the insurance coverage rate; take the United States as an example. From 2004 to 2007, when it is strongly pro-cyclical, the economic growth and income the average increased, and the insurance coverage rate increased slightly, but the insurance coverage through employers decreased; during 2007 to 2009, when the poor macro-economy or reverse cycle, the company's default rate increased. In order to reduce company costs, the company reduced employee insurance, especially when it encountered recession, the insurance coverage rate dropped significantly. For example, in 2001, the technology bubble and the 2008 subprime mortgage crisis caused an economic recession. The decline in income and the sharp rise in unemployment were accompanied by the loss of health insurance. Because they lost the insurance sponsored by their employers, there was a significant decline in insurance coverage through employers at this stage. There is less medical care than insurance (33), so the resulting unemployment will lead to a higher chance of death (34). A well-designed PCIS will stabilize the insurance industry and then benefit the social stability under a bad year.

The property-liability insurance business is obviously linked to the overall economic performance of the national economy (32, 35), especially when it comes to interest rates that are related to insurance pricing theory (36), insurance prices should reflect investment returns by discounting expected losses, so insurance prices are the result of discounting future losses. Any change in the interest rate causes a change in the premium because the insurance company invests the premium from the time the premiums receives to the time they pay the loss. However, interest rates are closely linked to the economic cycle, then the premiums are also affected by business cycles.

## Conclusion

The worldwide increasing frequency and intensity of disasters engender an upward trend in economic losses and in insured losses and thus damage the catastrophe insurance market. Many countries set up a public catastrophe insurance scheme (PCIS) to maintain the function of catastrophe insurance markets. Most literature about natural catastrophes focuses on risk management and the pricing of insurance policies. Most literature about insurance guaranty schemes discusses life insurances. Less analyzes the changes in stakeholders' interest (policyholders and insurers or equity holders) before and after the introduction of PCIS. The catastrophe insurance market with PCIS works well only when the interest is fair to all stakeholders.

This article develops a stakeholder framework with three cases of that the volume-based charges to the scheme may come from equity holders or policyholders or both. In the first case, insurers paying contributions incentivizes equity holders to lower their equity capital because insurers are not charged fairly. In the second case, policyholders paying contribution directly leads to that competitive pressure results in a race to the bottom and then forces the equity capital to the regulatory minimum equity level. The above two cases may cause adverse incentives with regard to the insurer's security level. The third case may be feasible because those policyholders pay insurers the same premium for full insurance and that insurers pay the PCIS the contribution based on insurers' insolvency risk. The value of the default put option measures the insolvency risk. Insurers can raise their equity capital to improve the insolvency risk, and then negotiate less contribution rate with the PCIS next year.

Various numerical illustrations focusing on sensitivity to PCIS' contribution rate show that the impacts of PCIS on stakeholders' interest are likely to be enormous such as equity holders' capital, insurers' default probabilities, and expected policyholders' deficits. In practice, most insurers' assets are invested in fixed income securities, and natural catastrophes bring in most insurers' liabilities of insured losses with cyclic up-trend characteristics. Therefore, the asset values are assumed to increase with a fixed rate in 1 year. The liability values are modeled to follow a mean-reverting process (CIR model) conditioned on the insured loss in the previous year. The numerical experiment is carried out via simulation and analytical form of default put option of insurers. The results show that the contribution rate (magnitude of volume-based levies) should be estimated carefully, not only with regard to PCIS's finance balance but also the resultant incentives and effects.

## Data Availability Statement

The raw data supporting the conclusions of this article will be made available by the authors, without undue reservation.

## Author Contributions

ZL: supervision, conceptualization, and resources. LZ: formal analysis, software, and visualization. M-CC: methodology and data curation. M-CW: writing—reviewing and editing. Y-CW: conceptualization, methodology, and writing—original draft. All authors contributed to the article and approved the submitted version.

## Funding

This study was supported by the National Social Science Foundation of China (Grant No. 19BJY090).

## Conflict of Interest

The authors declare that the research was conducted in the absence of any commercial or financial relationships that could be construed as a potential conflict of interest.

## Publisher's Note

All claims expressed in this article are solely those of the authors and do not necessarily represent those of their affiliated organizations, or those of the publisher, the editors and the reviewers. Any product that may be evaluated in this article, or claim that may be made by its manufacturer, is not guaranteed or endorsed by the publisher.
