# Supplementary material for: A Well-Designed Implement for Promoting Population Health and Property via Insurance
Source: Front Public Health. 2022 Jan 31;9:766003. doi: 10.3389/fpubh.2021.766003 (PMC8841659; doi:10.3389/fpubh.2021.766003)
Supplement: Supplementary file 1 [file Data_Sheet_1.docx]

**Appendix A**

The dynamics of insured loss follow a CIR model under physical measure (*P*):

$dL_{t}=a_{p}(b_{p}-L_{t})dt+\sigma_{X}\sqrt{L_{t}}dW_{L,\text{ }t}^{P}$, $L_{0}>0.$

In order to price the put option, the measure *P* is transferred in a risk neutral measure *Q* via Girsanov theorem.

$$dW_{L,\text{ }t}^{Q}=dW_{L,\text{ }t}^{P}-h\sqrt{L(t)}dt$$

where $a=a_{p}-h\sigma,\text{ }c\text{=}\frac{a_{p}b_{p}}{a_{p}-h\sigma_{X}}$，*h* denotes the risk adjustment factor. The CIR model under Q measure is written as:

$dL_{t}=a(b-L_{t})dt+\sigma_{X}\sqrt{L_{t}}dW_{L,\text{ }t}^{Q}$, $L_{0}>0.$

Given an given information, $Y_{T}:=c_{T}L_{T}$ follows a non-central Chi-Square distribution:

$$Y_{T}:=c_{T}L_{T}\sim\chi_{nc}^{2}(n,\delta_{T}),\text{ }g_{Y}(\varphi)=\frac{\exp\left(\frac{i\varphi\delta_{T}}{1-2i\varphi}\right)}{\left(1-2i\varphi\right)^{\frac{n}{2}}}$$

where $g_{Y}(\varphi)$ is a characteristic function of $Y$. $c_{T}=\frac{4a}{\sigma^{2}\left(1-\exp(-a(T-t))\right)},\text{ }n=\frac{4ab}{\sigma^{2}},\text{ }\delta_{T}=c_{T}L_{t}\exp(-a(T-t)),\text{ 2}ab\text{>}\sigma^{2}$

The risk adjustment factor *h* can be estimated by the following equation:

$L_{0}=E^{Q}[e^{-rT}L(T)]=\frac{e^{-rT}}{c}E^{Q}[cL(T)]=\frac{(n+\delta)e^{-rT}}{c}$.

The value of European put option is the expectation

Let *c* be any constant real number. $u(\cdot),\text{ }\delta(\cdot)\text{ and }f_{L}(x)$ are unit-step functions, Dirac-delta function, probability density function, respectively. Firstly, we derive the following expectation of indicator function:

$$E_{t}^{Q}[I_{\{d<Y_{T}\}}]=\int_{R} u(y-d)\cdot f_{Y}(y)dy$$

$$\text{=}\frac{1}{2\pi}\int_{R} \int_{R} u(y-d)\cdot e^{-i\varphi y}g_{Y}(\varphi)d\varphi dy$$

$$=\frac{1}{2\pi}\int_{R} \left[\int_{R} e^{-i\varphi y}\cdot u(y-d)dy\right]g_{Y}(\varphi)d\varphi$$

$$=\frac{1}{2\pi}\int_{R} \left[e^{-i\varphi d}\left(\pi\delta(\varphi)+\frac{1}{i\varphi}\right)\right]g_{Y}(\varphi)d\varphi$$

$$=\frac{1}{2\pi}\left[\pi+\int_{R} \frac{e^{-i\varphi d}g_{Y}(\varphi)}{i\varphi}d\varphi\right]$$

$$=\frac{1}{2}+\frac{1}{\pi}\mathrm{Re}\left(\int_{0}^{\infty} \frac{e^{-i\varphi d}g_{Y}(\varphi)}{i\varphi}d\varphi\right)$$

$$:=\Pi_{2,d}$$

Secondly, the probability density function can be written by$f_{Y}(y)=\frac{1}{2\pi}\int_{R} e^{-i\varphi y}\cdot g_{Y}(\varphi)d\varphi=\frac{1}{2\pi}\int_{R} \frac{e^{-i\varphi y}}{iy}\cdot g'_{Y}(\varphi)d\varphi.$

Finally, we derive the conditional expectation of loss distribution as follows:

$$E_{t}^{Q}[Y_{T}\cdot I_{\left\{d<Y_{T}\right\}}]=\int_{R} y\cdot u(y-d)\cdot f_{Y}(y)dy$$

$$=\frac{1}{2\pi}\int_{R} \int_{R} y\cdot u\left(y-d\right)\cdot\frac{e^{-i\varphi y}}{iy}\cdot g'_{Y}(\varphi)d\varphi dy$$

$$=\frac{1}{2\pi i}\int_{R} \left[\int_{R} e^{-i\varphi y}\cdot u(y-d)dy\right]\cdot g'_{Y}(\varphi)d\varphi$$

$$=\frac{1}{2\pi i}\int_{R} \left[e^{-i\varphi d}(\pi\delta(\varphi)+\frac{1}{i\varphi})\right]\cdot g'_{Y}(\varphi)d\varphi$$

$$=\frac{1}{2\pi i}\left[\pi g'_{Y}(0)-\int_{R} \frac{e^{-i\varphi d}g'_{Y}(\varphi)}{i\varphi}d\varphi\right]$$

$$=E_{t}^{Q}[Y_{T}]\left[\frac{1}{2}+\frac{1}{\pi E_{t}^{Q}[Y_{T}]}\mathrm{Re}\left(\int_{0}^{\infty} \frac{e^{-i\varphi d}g'_{Y}(\varphi)}{i\varphi}d\varphi\right)\right]$$

$$:=E_{t}^{Q}[Y_{T}]\cdot\Pi_{1,d}$$

where $g'_{Y}(0)=iE_{t}^{Q}[Y_{T}]=i(n+\delta_{T})$

The analytical formula of the European put option is:$P_{t}=Ke^{-r(T-t)}\cdot(\Pi_{2,0}-\Pi_{2,c_{T}K})-\frac{n+\delta_{T}}{c_{T}}e^{-r(T-t)}(\Pi_{1,0}-\Pi_{1,c_{T}K})$

$=Ke^{-r(T-t)}\cdot(1-\Pi_{2,c_{T}K})-\frac{n+\delta_{T}}{c_{T}}e^{-r(T-t)}(1-\Pi_{1,c_{T}K})$,

Since $Y_{T}$ is greater than zero because it is a random variable of non-central Chi-Square distribution, $\Pi_{2,0}=E_{t}^{Q}[I_{\{0<Y_{T}\}}]=1$ and $\Pi_{1,0}=E_{t}^{Q}[Y_{T}\cdot I_{\left\{0<Y_{T}\right\}}]/E_{t}^{Q}[Y_{T}]=1.$

**Appendix B**

Since $\text{H}_{0}^{*,E}-E_{0}^{*}=\text{PV}[(A_{t}+(L_{t}-A_{t})_{+}-C_{t}^{*}-L_{t})_{+}]-\text{PV}[(A_{1}-L_{1})_{+}]$, where $E_{0}^{*}=E_{0}=\text{PV}[(A_{t}-L_{t})_{+}]$, we derive the equation from following three conditions:

$$ifA_{t}-L_{t}<0,\text{ H}_{0}^{*,E}-E_{0}^{*}\text{=}0$$

$$if0\leq A_{t}-L_{t}\leq e^{\gamma t}C_{0}^{*},\text{H}_{0}^{*,E}-E_{0}^{*}=\text{PV}[(A_{t}-e^{\gamma t}C_{0}^{*}-L_{t})_{+}-(A_{t}-L_{t})]=-\text{PV}[(A_{t}-L_{t})]$$

$$ife^{\gamma t}C_{0}^{*}\leq A_{t}-L_{t},\text{H}_{0}^{*,E}-E_{0}^{*}=\text{PV}[(A_{t}-e^{\gamma t}C_{0}^{*}-L_{t})_{+}-(A_{t}-L_{t})]=-\text{PV}[e^{\gamma t}C_{0}^{*}]$$

To sum up, we have $\text{H}_{0}^{*,E}-E_{0}^{*}=-\text{PV}[\min(e^{\gamma t}C_{0}^{*}-(L_{t}-A_{t})_{+},(A_{t}-L_{t})_{+})]$.
